# Meetings of Societies

**Published:** 1904-06

**Authors:** 


### MEETINGS OF SOCIETIES.

### Bristol Medico-Chirurgical Society.

*March 9th, 1904.*

Mr. J. Paul Bush, C.M.G., President, in the Chair.

The following cases, specimens and lantern slides were shown:—

Mr. James Taylor: A series of lantern slides illustrating the value of the **X-rays in the diagnosis of Colles's fracture.**

The President: Lantern slides of **skiagraphs of some fractures and dislocations.**

Dr. Newman Neild and Dr. Theodore Fisher: Lantern slides illustrating a case of **congenital hypertrophic stenosis of the pylorus.**

Dr. E. C. Williams: (1) A case of **acquired syphilis** in a child aged 2 years; (2) a case of **argyria**; and (3) a case of **Graves's disease** in a girl aged 12 years.

Dr. P. Watson Williams: (1) A case of **incipient paralysis agitans**; (2) **frontal sinus empyema and ethmoidal cell disease** successfully treated by intranasal methods; (3) a case of **intra-laryngeal fibroma**; and (4) a case of **laryngeal tuberculosis** successfully treated by submucous injections.

Dr. W. Kenneth Wills: (1) A case of **lupus hypertrophicus** after light treatment; (2) a case of **keloid after using X-rays**; and (3) a case of **recurrent carcinomatous nodules** relieved by the X-rays.

Mr. A. W. Prichard: (1) A case of **dislocation of the lens, with coloboma of the choroid**; and (2) a case of **cysticercus of the vitreous.**

Mr. F. R. Cross: (1) Three cases of **paralysis** of the **third cranial nerve**; (2) three cases of **glaucoma**; (3) a case of **operation for double ptosis**; (4) a case of **retinitis proliferans**; and (5) a case of **astigmatism with very high cylinder lens.**

Dr. Clements Hailes: A case of **traumatic sclero-choroiditis.**

Dr. R. G. P. Lansdown: A case of **nævus of the leg.**

Dr. J. Lacy Firth: A patient free from recurrence after removal of a **cancer of the tongue and cancerous glands** on both sides of the neck four years previously.

Mr. T. Carwardine: (1) A patient after **gastro-jejunostomy for pyloric stenosis**; (2) a patient after operation for **cerebral abscess**; and (3) a patient after operation for **lateral sinus thrombosis.**

Mr. H. W. KENDALL: A **strangulated ovary in a hernial sac** of an infant.

Mr. H. F. MOLE: A **gangrenous ovary and Fallopian tube removed from a hernial sac** of an infant; (2) a **prostate removed by Freyer's method**; and (3) a case showing **healing of a large perforation of the membrana tympani**, with calcareous deposit.

Dr. E. W. HEY GROVES: (1) A case of **chancre of the tongue**; (2) a case of old **unreduced dislocation of the head** of the radius; (3) a case of **excision of the clavicle**; (4) a specimen of **carcinoma of the rectum**; and (5) **gallstones** from a man aged 26 years.

Dr. NEILD: A case of **acromegaly.**

Dr. W. H. C. NEWNHAM: (1) A **fibroid uterus with pyometra**; and (2) a **tubo-ovarian abscess.**

Dr. G. PARKER: (1) A case of **congenital heart disease**; and (2) cases of **injury to the upper extremity**, with lesions of the brachial plexus.

Dr. A. OGILVY: A case of **transposition of the viscera.**

Mr. A. L. FLEMMING: A case of **cervical ribs** (with skiagraph).

Mr. J. J. S. LUCAS: (1) A specimen of **cancer of the œsophagus simulating an aneurysm**; and (2) **multiple growths in the intestine,** with microscopic sections.

Mr. G. MUNRO SMITH: A photograph of symmetrical **abscesses in the calves,** causing an appearance simulating pseudo-hypertrophic paralysis.

Dr. H. WALDO: (1) Two cases of **lupus vulgaris** of the face in which no result had followed prolonged light treatment; (2) a case of **lichen ruber planus** of the neck and perineum; (3) a case of **universal alopecia**; (4) a case of **adenoma sebaceum** of the face; (5) a case of pityriasis rubra (dermatitis exfoliativa); (6) a case of **disseminated sclerosis**; and (7) a case of **mitral stenosis with presystolic** and **tricuspid systolic murmurs.**

Dr. F. H. EDGEWORTH: A case of **tuberculous disease followed by osteo-arthritis.**

Mr. C. H. WALKER: A case of **chronic ulcerative conjunctivitis** (? tuberculous) in a boy aged 16 years.

---

*April 13th, 1904.*

Mr. J. PAUL BUSH, C.M.G., President, in the chair.

Dr. STACK showed some specimens of **tubercular disease of the breast.** The microscopical preparations had been made by Mr. A. L. FLEMMING. The disease was formerly held to be very uncommon. Probably many specimens had been mistaken for scirrhus, or simply mastitis. The disease existed in two forms: in one there were scattered caseous foci; in the other there was diffused inflammation with very little evidence of caseation. Tubercular disease was seldom diagnosed as such, being usually mistaken for ordinary chronic mastitis. Two specimens of tubercular disease were shown, also one of adeno- or ring-carcinoma of the mamma, and one of cancer *en cuirasse*. —Mr. Munro Smith thought the first specimen was probably carcinoma, and he asked why the adenomatous tumour shown was considered to be adeno-carcinoma rather than adeno-fibroma.—Dr. E. W. Hey Groves mentioned a case in which he had removed a breast presenting numerous breaking-down sinuses, which proved to be tubercular. Some enlarged axillary glands which were present subsided later.

Mr. J. Paul Bush read notes on a **case of acute hemorrhagic pancreatitis.** He remarked that no signs of this disease were pathognomonic, and that a correct diagnosis was often only attained by a process of exclusion. His patient was a married woman of 28. She had been sent into the Bristol Royal Infirmary as a case of intestinal obstruction. She was very stout and looked older than she actually was. She had been suddenly seized with violent epigastric pain some hours before admission. Pressure increased the pain. There were frequent vomiting, a soft, small pulse of 120, and much sweating. Twelve hours later the vomiting still continued and the patient was worse. The abdomen was therefore opened, first below, then above the umbilicus. Fluid escaped, and after removing calculi from the gall-bladder a drainage-tube was inserted. On the tenth day some fluid which contained pancreatic ferments escaped through the upper wound. No sheaves of yellow crystals were obtained from the urine with Cammidge's test. The patient died, and the *post-mortem* showed extensive hemorrhage into the pancreas and extensively-diffused fat necrosis.—Mr. H. F. Mole alluded to a case of suppurative pancreatitis which had been treated in the Infirmary some years previously.—Mr. T. M. Carter alluded to a case of hemorrhagic pancreatitis he had had under his care some years previously. The patient had been a middle-aged man. Severe epigastric pain and profound collapse had been the most marked clinical features of the case. The heart sounds became so weak that they could scarcely be heard with an ordinary stethoscope. The patient had been very restless and mentally very alert. The rectum had shown a remarkable degree of ballooning, and obstruction of the bowels had been complete. The pancreas obtained from the patient had been shown by Mr. Firth at a previous meeting.—Dr. Waldo said he had seen six cases of the disease, two of which had recovered after operation. Glycosuria was rarely present. Chronic pancreatitis should be considered in cases in which severe jaundice persisted.—Mr. J. Lacy Firth confirmed the remarks Mr. Carter had made about his case. He himself had regarded that case as one of intestinal obstruction. Since then he had operated on a woman in the Hospital with symptoms suggesting a perforated gastric ulcer, and the operation had shown this case also to be one of acute pancreatitis, though not of a definitely hemorrhagic type. He had drained the pancreatic region. For ten days the patient's condition had improved, but about the sixteenth day she had succumbed. Lately he had operated upon a man with a history of attacks of pain accompanied with jaundice, suggesting biliary colic from gall stones. No gall stones were found at the operation, but the pancreas was felt to be enlarged and indurated, especially the head of pancreas. The man appeared to be improving as a result of draining the gall-bladder. The case seemed to be one of chronic indurative pancreatitis. There was no glycosuria, nor could Cammidge's crystals be found.—Mr. Bush added that there had been only very slight jaundice in his case, and a trace of albumen in the urine.

Dr. E. C. Williams read notes on a case of "**Blackwater Fever.**" (*Vide* p. 128.)—Dr. Fortescue-Brickdale had prepared microscopical specimens of the blood.—Mr. T. M. Carter alluded to a case of this fever he had had under treatment. The man had had an attack in Lagos. The attack the speaker had seen occurred about five weeks after the patient had returned to England. He had not given quinine, believing it not to be a serviceable drug for the disease.

Mr. G. Munro Smith discussed the **diagnosis of malignant tumours by the microscope,** and illustrated his remarks with lantern slides.

J. Lacy Firth.
H. F. Mole (*Hon. Sec.*).

---

